# Thrombospondin-1 contributes to slower aortic aneurysm growth by inhibiting maladaptive remodeling of extracellular matrix

**DOI:** 10.1042/CS20170275

**Published:** 2017-06-07

**Authors:** Mamoru Satoh, Takahito Nasu, Takuya Osaki, Sho Hitomi

**Affiliations:** 1 Division of Biomedical Information Analysis, Institute for Biomedical Sciences, Iwate Medical University, Japan; 2 Division of Cardiology, Department of Internal Medicine, Iwate Medical University, Japan

**Keywords:** Chemokine, cytokine, vascular inflammation

## Abstract

In this issue of *Clinical Science*, Krishna and colleagues describe recent work on thrombospondin-1 (TSP-1) maturation and its association with slower growth of aortic aneurysm in TSP-1 knockdown mouse models. The authors conclude that TSP-1 deficiency promotes maladaptive remodeling of the extracellular matrix (ECM) leading to accelerated aortic aneurysm progression. We comment on a causal relation between TSP-1 and the progression of aortic aneurysm.

The process of inflammation might be involved in the pathogenesis of broad ranges of cardiovascular diseases. Our previous reports have demonstrated that dysregulation of immune response against some cardiovascular risk factors, such as hyperlipidemia, hypertension, and diabetes, plays an important role in cardiovascular diseases including coronary artery disease and failing heart [1,2]. In particular, our previous report has shown that chronic aortic wall inflammation via up-regulation of tumor necrosis factor-α converting enzyme axis, which was one of metalloproteinase, was related to the pathogenesis of human abdominal aortic aneurysm (AAA) [3].

In this issue, Krishna and colleagues have focused on the relationship between serum levels of thrombospondin-1 (TSP-1) and growth of AAA in patients with AAA. In addition, the authors have shown the role of TSP-1 using apolipoprotein (ApoE) and *THBS1* gene (encoded TSP-1) double deficiency mouse model. The TSP family consists of five multimeric proteins containing multiple functional domains. They are calcium-binding glycoproteins and are secreted by a broad range of cell types including peripheral blood cells, vascular smooth muscle cells (VSMCs), endothelial cells, and fibroblasts [4]. The TSP family is divided into two subgroups (type A and B) according to structures of proteins [5]. The subgroup A TSPs (TSP-1 and TSP-2) have oligonucleotide domain, type I, II and III repeats, C-terminal domain, von Willebrand factor C type domain, and N-terminal domain [5,6]. On the other hand, the subgroup B TSPs (TSP-3–5) has oligonucleotide domain, type II and III repeats, C-terminal domain, and N-terminal domain [5,6]. TSPs are capable of binding and interacting with many cellular receptors and other extracellular matrix (ECM) molecules through their various domains [7]. Each TSP has different cellular distribution, transcriptional regulation, and functional capacities [8]. In particular, TSP-1 is the most studied TSP concerning cardiovascular diseases, being antiangiogenic and able to activate transforming growth factor (TGF)-β [6]. Recently, from some experimental studies, increased interest in cardiac TSP-1 expression has emerged [9–12]. TSP-1 null C57/BL6 mouse model has shown increased early hypertrophy and enhanced late dilation in response to pressure overload, although TSP-1 expression was increased in a mouse model of pressure overload because of transverse aortic constriction [9]. Mice lacking TSP-1 exhibit activity-associated increases in heart rate, central diastolic and mean arterial blood pressure, and a constant decrease in pulse pressure [10]. In addition, both TSP-1 and CD47 (TSP-1 receptor) null mice show exaggerated decreases in peripheral blood pressure and increased cardiac output and ejection fraction in response to nitric oxide [10]. Frangogiannis and colleagues have demonstrated Infarcted TSP-1 null mice exhibited sustained up-regulation of the chemokines monocyte chemoattractant protein-1, macrophage inflammatory protein (MCP)-1α, and interferon-γ-inducible protein-10/CXCL10 and the cytokines interleukin (IL)-1β, IL-6, and TGF-β, suggesting an enhanced and prolonged postinfarction inflammatory response [11]. It has also been reported to reduce neointima formation and decrease SMC activation due to decreased MMP-2 expression and reduced medial collagen deposition in TSP-1 and ApoE null mice [12]. The author’s previous report has shown that a novel antiangiogenic therapy (ABT-510 nonapeptide), antiangiogenic peptide mimetic of TSP-1, dramatically reduced adventitial angiogenesis, decreased entry of inflammatory cells, reduced collagen deposition, and reduced lumen surface area in the rat aortic interposition model of graft arteriosclerosis [13]. From these observations, TSP-1 may diminish maladaptive vascular16 remodeling and vascular inflammation accompanied with reduced inflammatory chemokine maturation.

Krishna and colleagues have examined a relationship between TSP-1 expression and vascular remodeling in patients with AAA and experimental models [14]. The authors have concluded that TSP-1 play an important role in chronic aortic wall inflammation and extracellular matrix remodeling in AAA. This report showed that high concentration of serum TSP-1 levels is related to slower growth of AAA in patients with AAA. Immunohistochemical analysis also showed that TSP-1 protein expression was relatively down-regulated in AAA body compared with AAA neck as non-aneurysm site. In agreement with these observations, it has been already reported that serum concentrations of TSP-1 were significantly lower in men with AAA (*n*=313, men) compared with non-aneurysmal controls (*n*=690) [15]. Adjusted multivariate analyses revealed significant negative associations between serum concentrations of TSP-1 with AAA [15].

An important insight provided by the authors is the effects of TSP-1 on aneurysm expansion using ApoE/TSP-1 double deficiency mouse model. This experimental study has shown that deficiency of ApoE/TSP-1 resulted in enlarged size of AAAs and increased atherosclerosis in response to angiotensin II (AngII) infusion. In addition, deficiency of ApoE/TSP-1 has enhanced extracellular matrix degradation, the systemic and aortic inflammation including macrophage inflammatory protein 1 (MCP-1) and IL-6 and matrix metalloproteinase 9 (MMP-9) expression in response to AngII. The authors have already reported an effect of a novel antiangiogenic therapy (ABT-510 nonapeptide), antiangiogenic peptide mimetic of TSP-1, on vascular inflammation and atherosclerosis [13]. These observations are in accordance with those by Moura et al. [12] who reported that the number of aortic infiltrating macrophages was higher in ApoE/TSP-1 double deficiency mice than in ApoE deficiency mice. In addition, ApoE/TSP-1 double deficiency mice showed an increase in elastin lumen degradation and accumulation of MMP-9 compared with ApoE deficient mice [12]. These observations have therefore suggested that TSP-1 has a beneficial effect of slowing AAA growth (Figure 1). However, Liu and colleagues have reported opposite results on effect of TSP-1 in AAA [16]. They found increased TSP-1 expression in the adventitia of human AAA [16]. TSP-1 expression was similarly induced in aneurysms of ApoE deficient mice treated with Ang-II [16]. In addition, mice lacking TSP-1 were more resistant to developing aneurysms in AAA models, and developed aneurysms exhibited less aortic expansion and inflammation than aneurysms in wild-type mice [16]. The authors explain about this opposite results that Liu and colleagues may be more relevant to human thoracic aortic aneurysm in which monogenetic mutations often lead to early presentation of disease in the absence of atherosclerosis [14]. Nevertheless, there are objections to this line of reasoning, so further examination is necessary.

**Figure 1:**
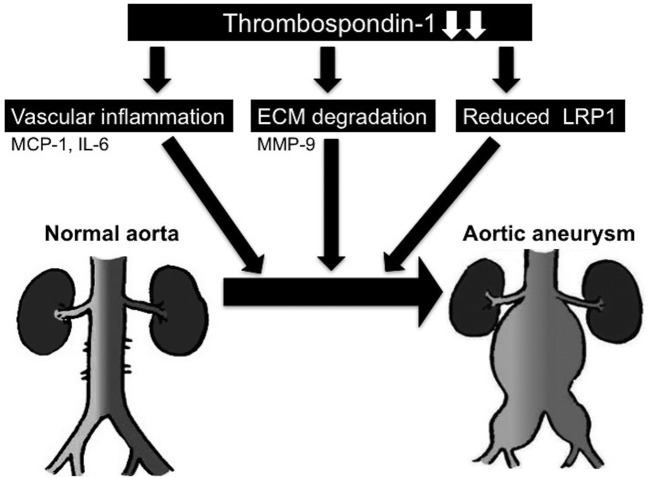
Effect of TSP-1 on pathology of AAA Proposed role of TSP-1 in the pathogenesis of aortic aneurysm. Repression of TSP-1 may enhance vascular inflammation via expression of cytokines, such as IL6 and MCP-1, ECM degradation via expression of MMP-9 and reduced LRP1. Finally, these pathways may induce expansion and aneurysm formation; LRP-1, low lipoprotein receptor-related protein 1; MCP-1, macrophage inflammatory protein 1; MMP-9, matrix metalloproteinase 9.

These results could have clinical implications for prevention or treatment of AAA. This study and their previous report [13] suggest that TSP-1 mimic agents could be used as novel therapeutic agents to the pathophysiology and expansion of AAA.

## Abbreviations

AAA: abdominal aortic aneurysm
Ang II: angiotensin II
ApoE: apolipoprotein
ECM: extracellular matrix
IL: interleukin
SMC: smooth muscle cell
TGF-β: transforming growth factor
TSP-1: thrombospondin-1
VSMC: vascular smooth muscle cell
